# Functionally enriched epigenetic clocks reveal tissue-specific discordant aging patterns in individuals with cancer

**DOI:** 10.1038/s43856-025-00739-4

**Published:** 2025-04-02

**Authors:** Chiara M. S. Herzog, Elisa Redl, James Barrett, Sepideh Aminzadeh-Gohari, Daniela D. Weber, Julia Tevini, Roland Lang, Barbara Kofler, Martin Widschwendter

**Affiliations:** 1 European Translational Oncology Prevention and Screening Institute, Universität Innsbruck, Innsbruck, Austria; 2 Institute for Biomedical Aging Research, Universität Innsbruck, Innsbruck, Austria; 3 Research Program for Receptor Biochemistry and Tumor Metabolism, Department of Pediatrics, University Hospital of the Paracelsus Medical University, Salzburg, Austria; 4 Department of Dermatology and Allergology, University Hospital of the Paracelsus Medical University, Salzburg, Austria; 5 Department of Women’s Cancer, UCL EGA Institute for Women’s Health, University College London, London, UK; 6 Department of Women’s and Children’s Health, Karolinska Institutet and Karolinska University Hospital, Stockholm, Sweden

**Keywords:** Breast cancer, Predictive markers, Cancer prevention, Cancer epigenetics

## Abstract

**Background:**

Aging is a key risk factor for many diseases, including cancer, and a better understanding of its underlying molecular mechanisms may help to prevent, delay, or treat age-related pathologies. Epigenetic alterations such as DNA methylation (DNAme) changes are a hallmark of aging and form the basis of so-called epigenetic clocks, yet their functional relevance and directionality in different organs during disease development is often unclear.

**Methods:**

Here, we link cell-specific age-related DNAme changes with three key hallmarks of aging and cancer (senescence, promoter methylation in genes associated with stem cell fate, and dysregulated proliferation) to comprehensively dissect their association with current and future cancer development, carcinogen exposure or preventive measures, and mortality using data in different organs from over 12,510 human and 105 mouse samples, benchmarking against existing epigenetic clocks.

**Results:**

Our findings offer insights into the association of functionally enriched groups of age-related DNAme changes with cancer, identify sites perturbed earliest during carcinogenesis, as well as those distinct between cancer and reprogramming that could inform strategies to prevent teratoma formation upon in vivo reprogramming. Surprisingly, both mouse and human data reveal accelerated aging in breast cancer tissue but decelerated epigenetic aging in some non-cancer surrogate samples from breast cancer patients, in particular cervical samples.

**Conclusions:**

This work provides evidence for discordant systemic tissue aging in breast cancer.

## Plain language summary

DNA contains genetic information about an individual. DNA is often chemically modified, a process called epigenetics, and it has been found that this occurs with distinct patterns during aging and in cancer cells. We looked at age-related DNA modification changes associated with the function of mouse and human cells. Our findings show that although cancer cells generally look epigenetically older than normal tissue, some non-cancerous cells from cancer patients show a younger epigenetic age. These findings highlight that aging may occur at different rates across the body and that cancer may be associated with deceleration of aging in some body parts. Our findings indicate that more research is needed to understand the role of these epigenetic modifications in aging as well as how aging rates across tissues mediate health and disease.

## Abstract (web summary)

Herzog et al. dissect functionally enriched groups of CpG associated with cellular senescence, proliferation and aging. Some non-cancer tissues from breast cancer patients show younger epigenetic ages than in controls, while breast cancer tissue looks generally older, providing evidence for discordant systemic aging in breast cancer.

## Introduction

Epigenetic changes are a hallmark of both aging^1^ and cancer^2^ and form the basis of many proposed biomarkers of aging, but often suffer from limited biological interpretability in the context of pathology. It is likely that not all age-related epigenetic changes are ‘created equal’: some may be causes of aging, some may be consequences of aging^3^, and depending on cell type and genomic location, aging may contribute to different cellular phenotypic states and functions. To better characterize epigenetic aging and its association with cancer as a key age-related disease, here we link cell type-specific age-related DNA methylation (DNAme) changes at cytosines followed by guanines (*5’—C—phosphate—G—3’*; thereafter referred to as CpGs) with three key hallmarks of aging and cancer: senescence, promoter methylation at genes associated with stem cell fate (polycomb group target genes, PCGTs), and dysregulated proliferation. These hallmarks are selected due to their known impact on the epigenome^4–6^ and important links to aging and carcinogenesis^4,6,7^.

The aim of this study is to comprehensively characterize the behavior of functionally enriched groups of age-related epigenetic features across cellular (e.g., reprogramming, senescence) and organismal contexts (e.g., cancer, exposures). We also investigate which DNAme alterations are (a) most strongly associated with carcinogenesis, (b) disrupted earliest during preneoplastic changes, and (c) whether age-related changes are differentially affecting diverse tissues in individuals with cancer, leveraging DNAme data from cancer tissue or at risk of developing cancer (normal adjacent tissue) and anatomically distant, non-invasive surrogate tissues (buccal, blood, or cervical samples). We focus on women’s cancers (breast, ovarian, endometrial and cervical cancer) due to the availability of a large dataset of surrogate samples from current cancer cases as well as the unique opportunity to explore DNAme changes during preneoplastic stages in the transition from intraepithelial cervical neoplasias (CIN, grades 1-3) to cervical cancer. Finally, we validate findings from observational human data with a mouse model of breast carcinogenesis^8^ with or without chemoprevention^9^, allowing for the study of DNAme changes in multiple organs from the same individual. Ultimately, the current study serves as a benchmark to explore the associations of functionally enriched groups of CpGs with exposures and age-related disease and mortality that may support future research into epigenetic clocks. Our findings reveal discordant tissue aging in breast cancer patients, with higher epigenetic age than in controls in breast tissue but lower epigenetic ages in cervical samples, and these findings validate in a mouse model. Taken together, these findings suggest that non-invasive samples have the potential to reflect disease risk in tissue at risk, but further research is required to contextualize the interpretation of epigenetic age in health and disease.

## Methods

### Datasets

This study leverages previously described datasets and a mouse methylation dataset generated for the purpose of this study. No separate IRB approval for human data was required as only existing data were leveraged. Each dataset was deposited by the original authors with approval of the respective IRBs, and no restrictions applied on data use with the exception of data from the National Survey on Health and Disease (NSHD). A full list of datasets, including accession numbers for GEO datasets, is provided in Supplementary Data 1. Briefly, we utilized the following datasets: GSE112812^10^, GSE112873^10^, GSE91069^6^, GSE82234^11^, GSE131280^12^, GSE116375^13^ (senescence); GSE197512^5^ (inhibition of proliferation); GSE72867^14^, GSE31848^15^ (fetal tissue methylation); GSE54848^16^ (Oct4, Sox2, Klf4, c-Myc reprogramming at various timepoints); GSE211668^17^ (cervical cancer); GSE236260 (mouse methylation study); TCGA methylation data; GSE184159^18^ (response to neoadjuvant chemotherapy); EGAS00001005078^19^ (cervical sample methylation from current or future cervical cancer); EGAS00001005055^20,21^ (cervical, blood, or buccal sample from current and future breast cancer cases), EGAS00001005045^22^ (cervical samples from ovarian cancer cases, excluding any samples with >0% inferred tumor material), EGAS00001005033^23^ (cervical samples from endometrial cancer cases, excluding any samples with >0% inferred tumor DNA), EGAS00001005070^24^ (breast tissue methylation from current or future breast cancer cases, or before and after mifepristone treatment). For NSHD data, we applied for data access for the purpose of this study and this was granted.

### Data preprocessing

With the exception of TCGA data, raw IDAT methylation files were preprocessed using the same standardized pipeline that has been previously described^20^ and is made available under https://www.github.com/chiaraherzog/eutopsQC. Crucially, this pipeline uses ssNoob, a single-sample normalization method suitable for incremental data processing and recommended for integration of data from multiple generations of Infinium arrays and experiment sets^25^, as required in the current study. For mouse data, we generated manifest and annotation R packages compatible with this pipeline, based on minfi and ChAMP, and make them publicly available on github (https://github.com/chiaraherzog/IlluminaMouseMethylationmanifest, https://github.com/chiaraherzog/IlluminaMouseMethylationanno.12.v1.mm10). Processed TCGA methylation data were accessed using TCGAbiolinks (version 2.28.3).

### Identification and evaluation of senescence-associated CpGs

Senescence-associated CpGs were identified using DNAme array data from senescence-induced and control cells (GSE112812, GSE112873, GSE116735, GSE131280, GSE82234, GSE91069)^6,10,12,13^ via CpG-level linear models of methylation beta value and type (senescent cell or control), additionally accounting for cell type and dataset (*n* = 89 samples total). The *p*-value histogram indicated an enrichment of sites significantly associated with senescence (Fig. S1a). More sites exhibited hypo- than hypermethylation with senescence (Fig. S1b). Autosomal CpGs with significant association with senescence after false discovery rate (FDR) adjustment (*p* < 0.05) were considered senescence-associated (*n* = 8278). We opted not to filter additionally based on delta beta differences as thresholds are often arbitrarily chosen, and we instead focused on the overall mean effect of all senescence-associated CpGs. A weighted mean of methylation levels according to directionality ( + 1 or -1) across these sites were associated with senescence within each dataset (Fig. S1c). Briefly, clock values were defined as follows:

$${clock}=\frac{{\sum }_{i}^{n}(w* \beta )}{n}$$

where *w*_i…n_ represent the directionality weights of the change of the CpGs i to n in the respective condition, $\beta$_i…n_ represent the methylation value of the respective CpG, and n represents the number of total CpGs in the clock. If a CpG exhibits higher methylation in senescence, it weights positively, whereas if it exhibits a lower methylation, it is weighted negatively to account for directionality. The final result is the sum of all these weighted contributions, divided by the total number of CpGs. The same approach was applied for other clocks below.

The senescence association was validated by assessment of correlation with *CDKN2A* (p16) mRNA expression levels in independent data from TCGA. This revealed that senescence-associated CpGs correlated positively with *CDNK2A* (p16) mRNA expression across multiple tissue types (Fig. S1d).

### Identification and evaluation of proliferation-associated CpGs

As a proxy for proliferation, we leveraged a dataset of dividing cells in which cumulative divisions had been tracked (GSE197545)^5^ and DNAme was profiled in proliferating or non-proliferating cells. CpG-level linear models of methylation beta value and type (reduced proliferation or control) were fitted, additionally accounting for original donor tissue and subexperiment/treatment (mitomycin C or serum withdrawal). The *p*-value histogram indicated an enrichment of sites significantly associated with senescence (Fig. S2a). Inhibition of proliferation induced more hypermethylation than hypomethylation (Fig. S2b). Autosomal CpGs with significant association with proliferation inhibition after FDR adjustment (*p* < 0.05) were considered proliferation-associated (*n* = 39,143). The weighted mean of methylation levels across these sites (accounting for directionality, as indicated above) was reduced upon inhibition of proliferation across multiple sub-experiments (Fig. S2c), and, importantly, correlated positively with *MKi67* (Ki67) mRNA expression in the independent TCGA dataset, one of the most commonly used markers for proliferation that is only expressed in the nucleus of proliferating cells^26^, across multiple tissue types (Fig. S2d).

### Identification of PCGT CpGs

Polycomb group target CpG sites were identified as previously described^27^. Briefly, we identified genes with single occupancy of at least one of SUZ12, EED, or H3K27me^28^ (*n* = 1591) and filtered CpGs within 200 bp of their transcription start site using the Illumina methylation array manifest (*n* = 1343). To obtain putative stem cell-associated genes, we filtered the list of CpGs to those that were unmethylated (mean beta below 0.2) in fetal tissues (GSE72867, GSE31848).

### Identification of age-related CpGs

Cell type-specific (epithelial or immune) or shared age-related CpG sites were identified by obtaining the Spearman’s rank correlation coefficient of DNAme levels for each CpG in immune cells (*n* = 211 blood samples from individuals aged 24–84, median age 53) or epithelial cells (buccal and cervical samples with <20% inferred immune cell proportion; *n* = 88 buccal samples and *n* = 122 cervical samples from individuals aged 20–81, median age 51) with chronological age. To obtain general, epithelial, or immune age-related CpGs, sites were filtered using the following criteria: General age-related CpGs (‘gen’) were required to be significantly correlated with age in both epithelial and immune cells after FDR adjustment (*p* < 0.05) and exhibit an absolute Spearman’s rank correlation coefficient >0.2 in both cell types. Epithelial- or immune-specific age-related CpGs (epi/imm) were required to be significantly correlated with age in epithelial or immune cells after FDR adjustment (*p* < 0.05), exhibit an absolute Spearman’s rank correlation coefficient >0.2 in the relevant cell type and <0.02 in the other cell type. Example sites passing these criteria are visualized in Fig. S3.

### Definition of overlapping signatures

The functionally enriched clocks of this study were defined by overlapping age-related CpG sites found to be located within 200 bp from the transcription start site of a gene occupied by PCGTs, sites significantly altered after inhibition of proliferation (either increased or reduced), or senescence, as follows:

*Senescence (SEN) CpGs:* Sites positively correlated with age that showed a hypermethylation in senescent cells, or sites negatively correlated with age that showed hypomethylation in senescent cells were overlapped.

*PCGT CpGs:* as PCGT CpGs were per definition unmethylated in fetal tissues, only those sites that positively correlated with age were considered for overlapping with PCGT sites.

*Proliferation (PRO) CpGs:* for the PRO+ signatures (increased dysregulated methylation with age), those sites sharing the same directionality with aging and increased proliferation were overlapped, while for the PRO- signatures (reduced methylation with age), sites with opposing directionalities were overlapped, indicative of reduced proliferation with age.

**Fig. 1:**
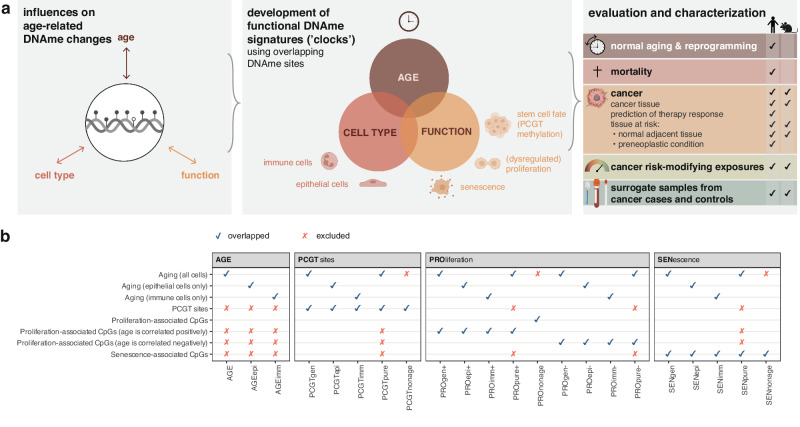
Development of cell type-specific functionally enriched DNA methylation (DNAme) aging clocks. **a** Age-related DNAme changes may be elicited, amongst other factors, by the overall aging process, cell type-specific processes, functional changes relating to aging, and the interplay between these three aspects. Here, we aimed to develop functionally enriched DNAme signatures indicative of one or more of these aspects by identifying overlapping CpGs, and evaluated these signatures in a variety of contexts. Discovery sets are annotated in **Supplementary Data 1**. **b** Overlaps utilized for development of CpG signatures. Icons from Biorender.com.

We also included functional signatures of CpGs not associated with age in either of the cell types (‘non age’) (Supplementary Data 2). Taken together, the above combinations yielded 22 distinct groups of CpGs altered with age either in specific cell types or shared between cells and associated with senescence, PCGT methylation, proliferation, or general aging alone (Fig. 1a, b, Supplementary Data 2, Fig. S4, 5).

### Assessment of sites in context of reprogramming, aging, cancer, and mortality

**Fig. 2:**
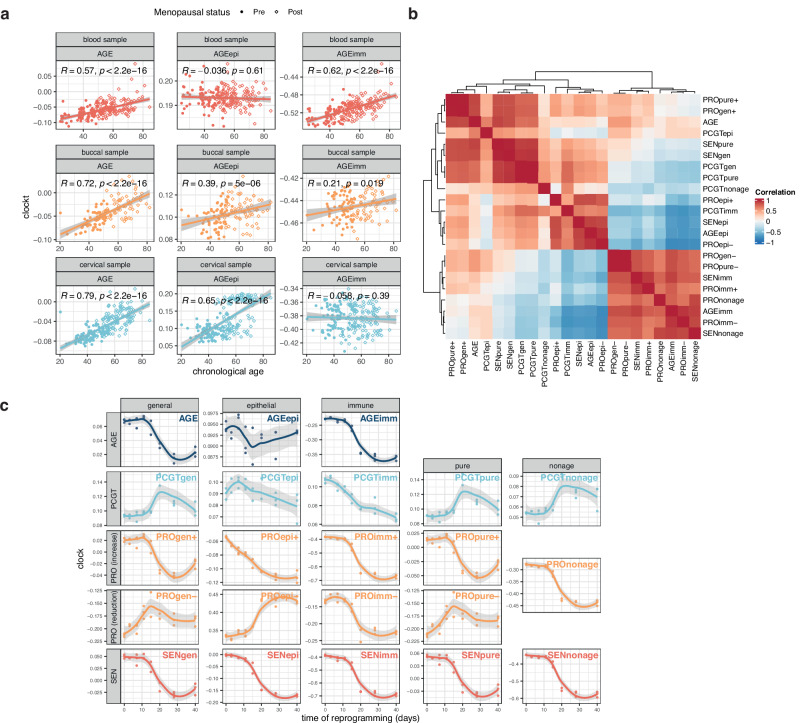
Initial assessment of cell type-specific functionally enriched DNA methylation (DNAme) aging clocks. **a** Visualization of overall shared, epithelial-specific, or immune-specific signature clocks (AGE, AGEepi, AGEimm) in blood, buccal or cervical samples derived from healthy women. Buccal and cervical samples with <20% inferred immune cell proportion were included. Sample shape indicates menopausal status (pre or post). Correlation coefficient is based on Spearman correlation. **b** Correlation of clocks in normal aging in samples shown in (**a**). Correlation coefficient is based on Spearman correlation. **c** Assessment of clocks over the course of treatment and reprogramming with the Yamanaka factors (OSK(M); Oct4, Sox2, Klf4, c-Myc). Source data for Fig. 2a-c are provided in Supplementary Data 8, 9, and 10, respectively. *N* = 208 biologically independent blood, *N* = 131 biologically independent buccal, and *N* = 214 biologically independent cervical samples in samples in (**a**, **b**). *N* = 21 biologically independent samples in (**c**).

**Fig. 3:**
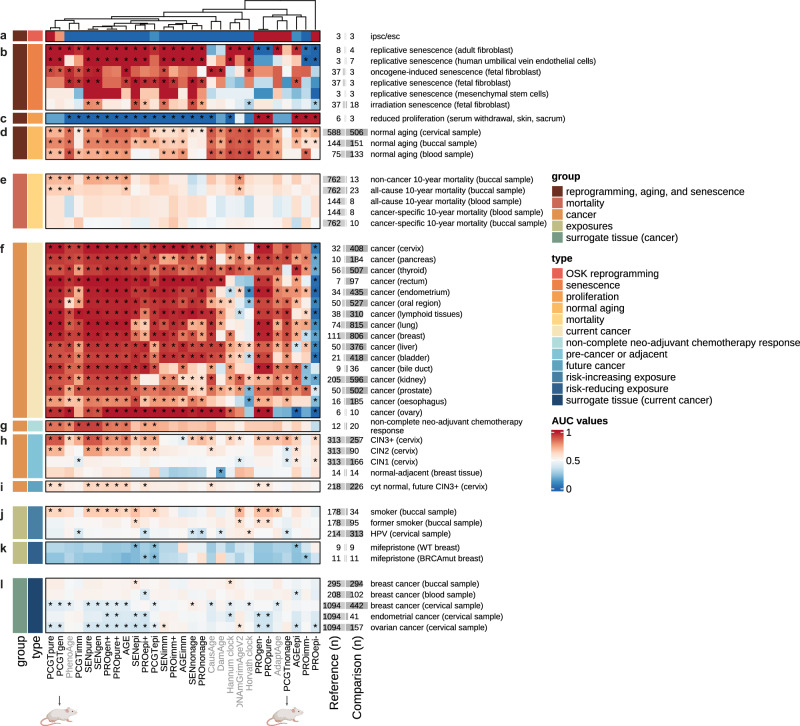
Changes in functionally enriched epigenetic clocks in response to reprogramming, senescence, proliferation, normal aging, cancer, exposures and mortality (l). Reprogramming is shown in (**a**), senescence in (**b**), proliferation in (**c**), normal aging in (**d**), cancer in (**e**–**k**), exposures in (**i**, **j**), and mortality in (**l**). Reference and comparison group details including accession numbers are provided in Supplementary Data 3. A total of 25 clocks were evaluated, 22 of these were defined as part of the current study (black column names) and 3 previously described (gray column names). The area under the curve (AUC) was computed compared to the respective reference group, accounting for changes in cell type composition and age where relevant (e.g., age was not adjusted in samples from the same individuals, see Methods). AUC values >0.5 indicate an increased epigenetic age compared to controls in the respective clock, while AUC values <0.5 indicate a lower clock value than the respective controls (see Supplementary Data 3): the directionality was maintained so that lower clock values than the respective reference group resulted in AUCs below 0.5 (shown in blue), whereas clock values higher than in the reference group are depicted by AUCs above 0.5 (red). Significant differences between the reference and comparison groups (confidence intervals not overlapping with 0.5) are indicated with an asterisk (*). Two clocks were later evaluated in a murine model of breast cancer (PCGTgen and PCGTnonage, Fig. 4), indicated using a mouse icon. Source Data for this figure are provided in Supplementary Data 11. Icons from Biorender.com.

Clocks for the 22 groups of CpGs were calculated for each dataset using the equation for weighted mean methylation above. Coefficients to calculate PhenoAge^29^, Horvath^30^, and Hannum^31^ epigenetic clocks were extracted from original manuscripts, respectively. GrimAge version 2^32^ and causality-enriched clocks (CausAge, DamAge, AdaptAge) recently described by Ying et al.^33^ were computed using the open-source library Biolearn (https://bio-learn.github.io)^34^. For the heatmaps in Figs. 2 and 3, the AUC was used as a standardized and easily visualizable measure for distinguishing between relative reference groups and comparison groups. By focusing on comparison to a relevant reference group within each datasets, we minimized the risk for batch effects. The directionality was kept fixed in order to be able to interpret older age clock values as increased, and younger clock values as decreased, relative to the respective reference group (Supplementary Data 3). Values for each clock were generally adjusted for chronological age and EpiDISH-inferred immune cell composition using linear models prior to comparison (i.e., the residuals were used), with the exception of the following comparisons: reprogramming (no adjustment); reduced/inhibited proliferation (no adjustment); samples from the same individuals before and after treatment with mifepristone (adjustment for immune cell proportion only). Any cervical samples from endometrial or ovarian cancer patients with tumor material contamination estimated to be >0% tumor DNA using the EpiDISH algorithm with endometrial cancer or high-grade serous ovarian cancer reference panels, as previously described^22,23^, were excluded. Wilcoxon tests were used to denote significance for AUC values that did not cross 0.5 (including their confidence intervals), and were FDR corrected. Heatmaps alongside reference/control sample numbers were visualized using the R ComplexHeatmap package.

#### Mouse study

**Fig. 4:**
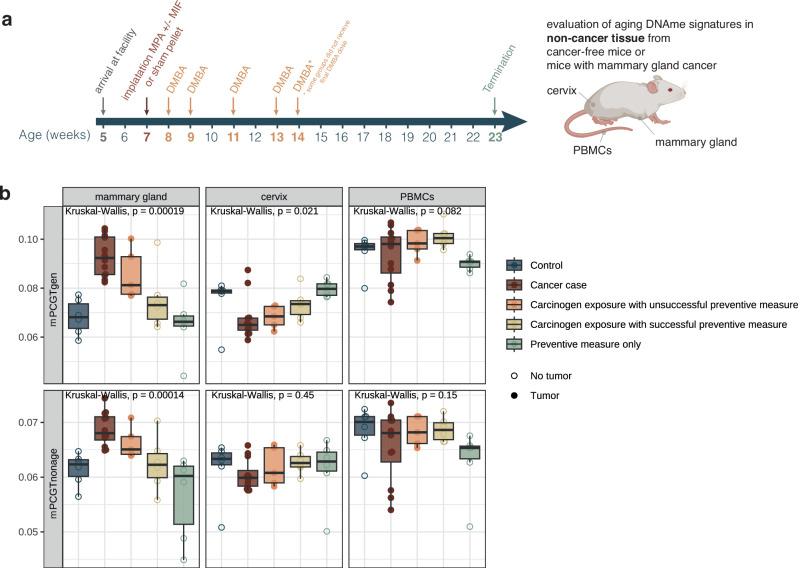
Mouse model of carcinogen exposure and prevention with multi-tissue methylation analysis. **a** Schematic of presentation of breast cancer mouse model and preventive treatment. Mice were exposed to a mammary gland cancer-inducing carcinogen (medroxyprogesterone acetate, MPA) via pellet implantation followed by repeated 7,12-dimethylbenz[a]anthracene (DMBA) oral gavage with or without a preventive measure (mifepristone, MIF; pellet implantation at same time as MPA). Appropriate vehicle controls were included. Unless animals were sacrificed due to large tumor sizes, termination occurred at 23 weeks of age. DNAme was analyzed in mammary glands free from cancer (equivalent to normal-adjacent tissue in human studies), cervix, and PBMCs from animals with or without mammary cancers as surrogate tissues. **b** Mouse PCGTgen (mPCGTgen) and PCGTnonage (mPCGTnonage) signatures in non-cancer tissue from the mammary gland, cervix, and PBMCs. Boxplots indicate median and interquartile ranges. Source data for Fig. 4b are provided in Supplementary Data 12. *N* = 35 biologically independent mammary gland, *N* = 35 biologically independent cervical, and N = 35 biologically independent PBMC samples in b. Icons from Biorender.com.

Animal experiments were performed at the animal facility of the Paracelsus Medical University Salzburg in accordance with the Austrian federal ministry of education, science and research (BMBWF), study approval No. 2021-0.236.530. We used a chemically-induced mouse model of breast cancer as described previously^8^. In brief, mammary tumors were induced in 7 week-old female BALB/c mice (Charles River) by subcutaneous implantation of 50 mg slow-release (90 days) medroxyprogesterone acetate (MPA) pellets (cat #NP-161, Innovative Research of America) and oral gavage administration of 1 mg of 7,12-dimethyl-benz(a)anthracene (DMBA) (cat #D3254, Sigma-Aldrich) dissolved in corn oil (cat #C8267, Sigma-Aldrich) once a week for a total of four to five times. On the same day of MPA pellet implantation, mice also received either placebo (*n* = 12) or 3 mg mifepristone (*n* = 11) (cat #M8046, Sigma-Aldrich) in form of slow-release (90 days) pellets (cat #NX-999, Innovative Research of America) by subcutaneous implantation (Fig. 4a). In the same manner, mice without mammary tumor induction (*n* = 12) received placebo pellets (cat #NC-111, Innovative Research of America) and oral gavage containing corn oil as the DMBA corresponding vehicle. Mice were randomly assigned to experimental groups to ensure equal distribution and comparable body weight across groups. The experiments were conducted in two runs, separated by 1 week, with all mice being 7 weeks old at the time of pellet implantation surgery. For each run, 50% of the mice in the MPA pellet/placebo pellet and MPA pellet/mifepristone pellet groups underwent pellet implantation surgery on day 1, while the remaining 50% underwent surgery on day 2. Surgery was performed alternately between the two groups. Similarly, surgeries for the MPA placebo pellet/mifepristone placebo pellet and MPA placebo pellet/mifepristone pellet groups were performed on day 3, alternating between groups. No specific method was used to generate a randomization sequence, and experimenters were not blinded to the treatment group. No specific strategies were employed to explicitly control for order of treatments and measurements, or animal and cage location.

Mice were euthanized when they reached 23 weeks of age or earlier if the size of the mammary tumors exceeded 200 mm^3^, as estimated by palpation and measuring by a caliper using the formula: width × height × length / 2. All 12 placebo-treated mice in the MPA/DMBA group developed tumors before the experiment ended at week 23, with an average onset of 82 days after pellet implantation, indicating unsuccessful treatment. In contrast, 6 out of 11 mifepristone-treated mice did not develop tumors by the end of the 23-week period, representing successful treatment. The remaining 5 mifepristone-treated mice developed tumors, with an average onset of 85 days after pellet implantation. Placebo-treated mice were euthanized when their tumors reached an average size of 250 mm² (range: 70–424 mm²). Mifepristone-treated mice that developed tumors were euthanized at an average tumor size of 204 mm² (range: 72–264 mm²). All samples, including tissues (cancer-free mammary glands) and PBMCs obtained by heart puncture, were frozen in liquid nitrogen and then stored at -80 °C for DNAme analysis. Samples were processed in a blinded manner during laboratory processing and DNAme data generation.

For assessment of BALB/c mouse age-related DNAme sites, untreated mice were sacrificed at different timepoints (ages 86, 122, 150, 180, 210, 240, 270, 309, 339 days). Adipose, breast, cervix, kidney, liver, lung, lymph node, oviduct, and spleen tissue and PBMCs were frozen in liquid nitrogen and stored at -80 °C for DNAme analysis.

### Methylation profiling for mouse samples

Extracted DNA from mouse samples was normalized to 12.5 ng/µL in nuclease-free water, and 250 ng total DNA was bisulfite-modified using the EZ-96 DNA Methylation-Lightning Automation kit (#D5049, Zymo Research Corp, cat) on a Tecan Fluent® 480 liquid handling platform. Bisulfite-modified DNA was eluted in 15 µL of nuclease-free water and 8 µL of modified DNA were subjected to methylation analysis on the Illumina Infinium Mouse Methylation BeadChip microarray (Illumina, CA, USA) at the European Translational Oncology Prevention and Screening (EUTOPS) Institute in Zams, Tyrol, Austria, according to the manufacturer’s standard protocol. Processed BeadChips were imaged within 24 h on the Illumina iScan™-System. Raw data were processed using the eutopsQC package (https://www.github.com/chiaraherzog/eutopsQC). Briefly, raw methylation microarray data were loaded using the R package minfi, version 1.46, with minor modifications to allow for annotation of the mouse array that is not by default included in the minfi package. For this, we generated annotation (https://www.github.com/chiaraherzog/IlluminaMouseMethylationanno.12.v1.mm10) and manifest (https://www.github.com/chiaraherzog/IlluminaMouseMethylationmanifest) R packages based on the array information provided by Illumina. Any samples with median methylated and unmethylated intensities <9.5 were removed. Any probes with a detection *p*-value > 0.01 were regarded as failed. Any samples with >10% failed probes, and any probes with >10% failure rate were removed from the dataset. A total of six samples were removed (one oviduct and two spleen samples from healthy mice in the discovery set, and one breast and two spleen samples from MPA/DMBA-exposed mice in the discovery set). Background intensity correction and dye bias correction was performed using the minfi single sample preprocessNoob function. Probe bias correction was performed using the beta mixture quantile normalization (BMIQ) algorithm in the ChAMP package, version 2.30.0, with a modification to allow for processing of the mouse array. The top 30,000 variable probes (ranked by standard deviation) were used in a principal component analysis. Correlation or Kruskal Wallis tests were performed in order to identify any anomalous associations between plate, sentrix position, date of array processing, date of DNA isolation, sampling date, DNA concentration, and the top ten principal components. No issues were identified. A PCA plot of the top 30,000 variable CpGs across diverse tissues from the aging set is shown in Fig. S6.

### Definition of mouse aging signatures

For the mouse, no matched expression and methylation data were available and hence we were not able to define equivalent PRO or SEN clocks. We defined mouse PCGTgen (mPCGTgen) and mouse PCGTnonage (mPCGTnonage) clocks as follows: (1) PCGT genes were identified using the same list as for human data (see “Identification of PCGT CpGs” above), converting human gene symbols into murine orthologs using the R package babelgene (version 22.9); (2) mouse PCGT CpGs were identified by filtering the annotation of the Illumina Methylation mouse array for murine PCGT gene symbol orthologs, keeping only genes located within 200 bp of a transcription start site (TSS200) and unmethylated in fetal mouse tissue (mean beta <0.2 across *n* = 41 fetal samples from the mouse methylation atlas (NCBI GEO GSE184410), including fetal brain (*n* = 11), intestine (*n* = 9), limb (*n* = 10), and liver (*n* = 11). Mouse CpGs were located in genes that exhibited a higher propensity score than background in a previous study by Liu. et al. describing the prediction of murine PCGTs^35^ (Fig. S7b); (3) sites correlated with age (cor > 0.2, *p*.adjust < 0.05) or not correlated with age ( | cor | <0.2) across several mouse tissues (*n* = 87), including breast, oviduct, cervix, adipose tissue, blood, liver, spleen, and others, were identified, correcting for tissue type. Any sites on X, Y, or MT chromosomes were excluded; (4) mPCGTgen CpGs were defined as mouse PCGT sites correlated with age, while mPCGTnonage sites were defined as mouse PCGT sites not correlated with age.

### Statistics and reproducibility

Our analysis leveraged large sample sizes and standardized scripts. No technical replicates were included. Sample sizes are indicated in the individual figures; in total, DNA methylation data from over 12,000 human and mouse samples were leveraged for this study. All code to reproduce analyses is available publicly^36^.

### Reporting summary

Further information on research design is available in the Nature Portfolio Reporting Summary linked to this article.

## Results

### Definition of functionally enriched aging signatures

To dissect epigenetic aging by function and cell type we defined 22 groups of CpGs by correlating them with age in epithelial (epi), immune (imm), or both cell types (general) and overlapping them with loci associated with senescence (SEN), PCGT methylation (PCGT), or dysregulated proliferation (PRO) **(**Fig. 1a, b; Supplementary Data 1; see Methods section). Epithelial and immune cell types were chosen as they represent two of the most common cell types in surrogate samples and highly relevant for cancer development, in particular women’s cancers such as breast, ovarian, and endometrial cancer. Senescence is a heterogeneous process^37^, and we therefore combined multiple previously-described datasets^6,10,12,13^ of control cells and cells in which senescence had been induced via irradiation, replicative senescence, or oncogenes, totaling *n* = 89 samples. As we were agnostic as to whether dysregulation of proliferation was associated with increased or reduced proliferation (or both), we defined both types: PRO+ sets of CpGs were positively associated with both age and proliferation (i.e., higher values indicate *increased* proliferation), whereas PRO- were positively associated with age but negatively associated with proliferation (i.e., higher values indicate *reduced* proliferation). Sites associated with aging but not any of the functional features were termed ‘AGE’, whereas CpG sites associated with function but not aging were termed ‘nonage’. Sites associated with aging and exclusively one feature but not others were termed pure (e.g., PCGTpure contains age-related CpGs purely associated with PCGTs but excludes any CpGs associated with senescence or proliferation) (Fig. 1a, b; Figs. S1, S2, S5). A full list of age-related CpGs and their genetic location is provided in Supplementary Data 4, while CpGs associated with senescence, proliferation, and PCGTs are provided in Tables Supplementary Data 5–7. These groups of CpG were functionally enriched and associated, amongst others, with mRNA expression levels of senescence- or proliferation-associated genes (Figs. S1, S2).

The association of methylation levels in these groups of CpGs with various factors, including carcinogenesis, mortality, or prognosis of pathological response in cancer therapy, was evaluated within each dataset using a quantitative score, derived from computing the weighted mean for each group of CpGs. We deliberately opted not to train predictors using penalized regression or other tools, e.g., to predict chronological age, to avoid optimizing a predictor for chronological age that carries increasingly less information about biological age with optimized performance to predict chronological age. For simplicity, the quantitative measures we defined are thereafter referred to as clocks (e.g., AGEepi clock).

Visualization of the clocks confirmed the cell type-specificity (Fig. 2a; Fig. S5). Notably, the AGEepi clock exhibited a dependence on menopausal status in cervical samples, as previously observed^38^. We note that features correlated with aging exclusively in one cell fraction may not specifically exhibit higher correlation in that tissue compared with generally age-correlated features (e.g., AGE exhibits higher correlation with chronological age in epithelial fractions than AGEepi, as previously observed in a similar cell-specific clock^38^). A correlation matrix of the 22 clocks in blood, buccal, and cervical samples suggested, in line with our initial hypothesis, the existence of different age-related modules of CpGs (Fig. 2b), although these did not necessarily cluster by cell type or function.

### Functionally enriched age-related sites exhibit divergent changes upon reprogramming

We initially confirmed the general association of the relevant clocks with age (Fig. 2a, Fig. S5), benchmarking against commonly used but diverse epigenetic clocks that were developed using different algorithms and training strategies, including chronological age (Hannum clock^31^, Horvath clock^30^) and healthspan (PhenoAge^29^). We also compared our clocks against GrimAge version 2^32^, one of the to date strongest predictors of mortality, and recently described causality-enriched epigenetic clocks^33^, both made openly available in the open-source library Biolearn^34^. These clocks were chosen as they are most frequently used, exhibit associations with mortality or healthspan, or are causality-enriched for age-related sites. The integrative analysis of existing biomarkers with with clocks developed during the current study may inform on similarities and differences with these specific biomarkers. Generally speaking, previously developed biomarkers correlated better with age in immune than epithelial cells, in line with the predominant development of epigenetic biomarkers in blood samples that primarily consist of immune cells. In particular PhenoAge exhibited a much worse correlation with chronological age in the epithelial fraction than in the immune fraction (0.48 versus 0.89, respectively).

We next aimed to understand the impact of reprogramming, which has previously been described to reverse many aspects of epigenetic aging^39^, on the clocks developed as part of this study (Fig. 2c), leveraging a previously described dataset of fibroblasts longitudinally sampled during OSKM (Oct-4, Sox2, Klf4, c-Myc) reprogramming (GSE54848)^16^. As expected, the majority of age-related DNAme features were generally reversed during reprogramming (14/22), although not all clocks shared this behavior, notably those associated with stem cell gene promoters (PCGTs). Several PCGT clocks showed a transient ‘spike’ at the time when other clocks indicated rejuvenation (day 20), and interestingly, sites associated with decreased age-related proliferation in epithelial cells (PROepi-) showed an apparent increased age after reprogramming. Our findings complement a previous study evaluating distinct modules of existing epigenetic clocks over the course of reprogramming^40^, and may contribute to better understanding of epigenetic dynamics of reprogramming. A more detailed investigation of cell type specificity in the context of reprogramming is limited by the fact that most donor-derived reprogramming studies to date have been conducted in fibroblasts, and future studies in other cell types should be encouraged as they may shed more light on potentially diverging responses to reprogramming.

### Senescence and normal aging increase epigenetic age

Figure 3 summarizes the evaluation of the 22 clocks defined in this work, again benchmarking against Hannum, Horvath, PhenoAge, GrimAge (V2) and causality-enriched clocks. Figure 3 shows values in these clocks in normal aging and reprogramming, mortality, cancer, and exposures. In each setting and row, samples were compared to a relevant reference within the same dataset (see Supplementary Data 3 for reference and comparison group information, sample size and accession numbers) and the area under the curve (AUC) was computed and visualized using a heatmap after accounting for chronological age (where applicable) and immune cell proportion. Clock values were compared within each dataset. AUCs at 0.5 indicated no distinction between cases and control, whereas AUCs of 1 and 0 indicated perfect distinction in the expected and unexpected direction, respectively (see Fig. S7a; Supplementary Movies 1 and 2). Asterisks indicate FDR-corrected *p*-values for Wilcoxon tests for any AUCs whose confidence intervals did not cross 0.5. Clocks generally exhibited reduced values in induced pluripotent or embryonic stem cells compared to their respective controls (donor fibroblasts, Fig. 3a) although this did not retain statistical significance after false discovery rate correction, likely due to the limited number of samples (n = 3 per group), whereas they were consistently elevated in senescent cells relative to control cells, regardless of induction mode (Fig. 3b). Inhibition of proliferation appeared to reduce cellular aging (as represented by the various clocks) (Fig. 3c). In line with previous observations^41^, normal aging - comparing pre- and post-menopausal samples for simplicity - exhibited the opposite behavior to reprogramming in the majority of the clocks, although not all clocks exhibited this behavior, e.g. PCGTpure (Fig. 3d). Our findings together suggest that many of the clocks, as expected (and per definition), are elevated with increasing age and senescence, and reduced with reprogramming.

### Functionally enriched clocks are equally predictive of 10 year mortality as clocks trained to predict mortality

Epigenetic age acceleration has previously been shown to be predictive of mortality^42^, and specific clocks predicting mortality or lifespan have been developed (e.g.^29,43,44^). Thus, we evaluated whether the defined functionally enriched clocks were predictive of all-cause mortality over 10 years using a small dataset of buccal and blood samples derived from a birth cohort (Fig. 3e) and compared them to commonly used epigenetic clocks predicting chronological age (Hannum^31^ and Horvath^30^ clocks) mortality/healthspan (PhenoAge^29^, GrimAge version 2^32^), and causality-enriched clocks^33^. Several of the age-related clocks exhibited significant differences between individuals who died within 10 years of sample collection compared to those who did not, both for all- and cause-specific mortality (cancer versus other), but interestingly, the differences were only apparent in buccal and not blood samples. The AUC for all-cause mortality was significantly higher for some of the functionally enriched clocks than for Horvath and Hannum clocks, neither of which showed an AUC significantly different from 0.5, and some even exhibited a higher AUC than PhenoAge (AUCs AGE: 0.66 [95% CI: 0.53–0.79], PhenoAge: 0.65 [0.52–0.78]), an epigenetic clock trained to predict lifespan, albeit sample numbers were small. GrimAge (V2) retained the highest AUC for predicting mortality, although surprisingly more so in buccal than blood samples (AUC: 0.68 [0.55–0.80]).

We note that the AUC may be an imperfect outcome for estimating mortality as it leverages a binary outcome without considering more granular time-to-event, and hazard ratios may be preferable. For the current dataset, no detailed information on time-to-death was available which precluded a more in-depth assessment of such scores.

### Cancer tissue exhibits accelerated aging compared to control tissue in most clocks

We next assessed the clocks in the context of cancer, a key age-related disease. As expected, many of the clocks were profoundly altered in cancer tissue relative to normal (adjacent) control tissue (Fig. 3f). The majority of clocks showed values in line with accelerated aging across multiple features (proliferation, senescence, PCGT methylation) compared to control tissue after adjusting for chronological age and sample immune cell proportion to account for cellular composition. Our data are consistent with previous findings that suggest cancer tissues exhibit accelerated aging features^30,45,46^. Interestingly, clocks associated with aging in epithelial cells that are negatively associated with proliferation (PROepi-, higher value indicating reduced proliferation) were consistently reduced in cancer tissue, indicating that cancer tissues exhibited a younger epithelial age with regards to proliferation. Notably, the Horvath clock also showed a younger epigenetic age compared to normal control tissue in several cancer types when compared within tissues, whereas Hannum and PhenoAge clocks exhibited similar area under the curve values as many of the accelerated clocks. Our analysis revealed that there exist both shared (e.g., PCGTpure, PROgen-, PROpure-) and diverging features (e.g., PROepi-, SENpure, SENgen, AGE) between cancer and reprogramming **(**Fig. 3a, f**)**. Information on sites diverging between cancer and reprogrammed cells may be the basis for further investigations to understand and ultimately prevent carcinogenesis risk during reprogramming.

### Aging signatures are prognostic of complete pathological response to neoadjuvant chemotherapy

Biomarkers of aging, such as epigenetic clocks, may have several uses, inter alia as prognostic biomarkers^3^. Multiple clocks exhibited significantly higher values in breast tissue collected prior to treatment from individuals who did not, or only partially, respond to neoadjuvant chemotherapy compared to those who exhibited a complete response (Fig. 3g), indicating epigenetic features of aging could offer a prognostic value.

### Samples collected during carcinogenesis reveal early epigenomic changes and compensatory pathways

Established cancers often show considerable (epi)genomic reorganization and thus do not offer insights into early carcinogenic events. To further investigate ongoing processes at these earlier stages that have previously been described to experience epigenetic drift^47^, we assessed our functionally enriched aging signatures in cervical samples from women with precancerous cervical intraepithelial neoplasia (CIN) stages 1 to 3+ relative to human papillomavirus positive (HPV)+ but cytologically normal cervical samples (Fig. 3h). Surprisingly, dysregulation of age- and cancer-associated clocks was apparent as early as CIN1 (e.g., elevation of AGEepi), and the majority of features apparent in CIN2 and CIN3+ overlapped with those observed in cervical cancer, albeit less pronounced. Interestingly, a few signatures exhibited opposite directionality between CIN1/CIN2 and CIN3+ (PCGTnonage, PCGTimm), indicating possible compensatory mechanisms during early preneoplastic stages. Cytologically normal samples from women that went on to develop CIN3+ up to 4 years after sample donation likewise already showed cancer-related changes in the signatures that could possibly help to predict their risk and enable more targeted screening (e.g., PCGTpure, PCGTgen) (Fig. 3i). Normal breast tissue adjacent to cancer shared clock features with both CIN1 and CIN2, in line with an epigenetic defect observed in precancerous conditions^48^, but these observations were not significant, possibly due to small sample size (*n* = 14 per group; Fig. 3h).

### Exposures modifying cancer risk also modify epigenetic age in relevant tissues

We assessed whether age-related clocks were modifiable by exposures that either increase or decrease cancer risk. Risk-increasing exposures mirrored changes in cancer tissue, whereas risk-reducing treatments inverted the effect (Fig. 3j, k): clocks in buccal samples from smokers exhibited similar patterns as those observed in cancer tissue of smoking-related cancers (e.g., esophagus, lung). Conversely, the breast cancer risk-reducing selective progesterone receptor modulator mifepristone significantly reduced cancer-related clocks in the breast compared to pre-treatment in a paired sample analysis^24^. HPV infection in cervical samples appeared to elicit certain changes similar to pre-cancer stages (CIN1), e.g., PCGTnonage and PCGTimm, in line with the finding that HPV is a key driver of cervical intraepithelial neoplasias^49^. However, our findings indicate that the changes were not similar to those in established cancers and instead might reflect previously identified compensatory mechanisms, such as apoptosis^50^, and the fact that a majority of HPV infections resolves without carcinogenesis^49^.

### Surrogate cervical samples from cancer cases exhibit inverse aging profiles compared with cancer tissue, and show a reduced epigenetic age compared to controls, while buccal samples mirror changes in cancer tissue

While changes in tissue at risk of developing cancer are informative, they may not be practical for routine or repeat monitoring. Instead, non-invasively collected surrogate samples, such as blood, buccal, or cervical samples may be more suitable. We therefore evaluated aging-related changes in a sample collection consisting of blood, buccal or cervical samples from women with breast, ovarian, or endometrial cancer and age-matched controls (Fig. 3l). We observed changes in age-related DNAme clocks even in these anatomically distant surrogate samples. In particular, cervical samples from breast, ovarian, and endometrial cancer cases (samples with 0% inferred tumor material only) all exhibited similar clock patterns, indicative of a systemic effect predisposing to cancer, akin to a field defect albeit in expressing itself in an opposing directionality compared to cancer tissue. The clocks generally exhibited the opposite directionality to changes observed in cancer tissue: for instance, PROgen+ was generally elevated in breast cancer tissue compared to normal breast tissue (Fig. 3f), whereas it was reduced in cervical samples from breast cancer cases compared to controls (Fig. 3l). In buccal and blood samples, differences from controls were less pronounced (Fig. 3l**)**. Interestingly, buccal samples shared the directionality with changes in cancer tissue and indicated a significantly higher epigenetic age compared with controls in the Hannum and SENepi clocks (Fig. 3l). These findings are consistent with findings from our recent study that shows buccal epigenetic changes mirror those in the breast, with cervical changes appear to exhibit opposing directionality^51^. These findings suggest that both tissues may have potential to detect the presence of breast cancer, but buccal samples are more aligned with changes observed in breast cancer tissue than cervical samples.

### A mouse model of breast cancer and prevention validates human findings of inverse relationship between epigenetic age in cancer and surrogate tissue

The finding of an inverse association of epigenetic aging in cancer tissue or tissue at risk (i.e., acceleration) and cervical surrogate tissue (deceleration compared to controls) was intriguing. Thus, we validated our findings with DNAme analysis in a chemically-induced mouse model of breast cancer^8^ with or without a preventive exposure (mifepristone) (Fig. 4a). As representative functionally enriched clocks, we defined mouse PCGTgen (mPCGTgen) and mouse PCGTnonage (mPCGTnonage) clocks (refer to Methods section for details; Fig. S8). These clocks were chosen based on their feasibility for transfer to mice, leveraging the availability of existing data and the evolutionary conservation of PCGT genes, suggesting their applicability in murine models. We applied them to non-cancer tissue derived from the mammary gland, cervix, and peripheral blood derived mononuclear cells (PBMCs) from mice with or without a tumor. These data confirmed our findings previously observed in humans: normal mammary glands from animals with a cancer (i.e., cancer in another mammary gland) exhibited increased mPCGTgen/mPCGTnonage (Fig. 4b). Preventive mifepristone exposure reduced values of these clocks, and values exhibited a greater reduction in animals without a tumor compared to those with a tumor, suggesting that the epigenetic features may be associated with the effectiveness of the treatment and could be used to measure the effects thereof. Mifepristone alone (no carcinogen) did not alter values of these clocks compared to controls (no carcinogen, no mifepristone). In the cervix, this pattern was inverted: mPCGTgen/mPCGTnonage were both reduced in cervical tissue from mice with mammary gland cancer. As previously observed in humans, blood DNAme did not exhibit significant changes in aging clocks compared to controls, although interestingly, both clocks appeared to be decelerated by mifepristone exposure alone.

## Discussion

Previous studies have already provided highly interesting data evaluating the association of ‘epigenetic clocks’ correlated with chronological age (e.g., Horvath^30^, Hannum clocks^31^), PCGT/PRC2-related^27,52^ or other clocks and their association with cancer risk both in the tissue at risk or other healthy tissues. However, to our knowledge, our study is the first to comprehensively investigate functionally enriched modules of age-related and cell type-specific DNAme sites associated with senescence, proliferation, and stem cell fate, and their association with current and future cancer in tissue at risk or surrogate samples. We define groups of CpGs associated with these hallmarks, and independently validate their association with mRNA expression levels of senescence- and proliferation-associated genes (Figs. S1, S2). Our study also evaluates the impact of risk-increasing or -reducing exposures on age-associated features, and evaluates the predictive power of functional modules for response to chemotherapy and all-cause mortality. In contrast to previous studies (e.g., CellDRIFT^52^), our study did not involve training predictors for cumulative population doubling, chronological age, time-to-death, or cancer, but instead used mean weighted methylation values of subgroups of CpGs moderately correlated with chronological age and associated with senescence, dysregulated proliferation, or stem cell fate, to construct functionally-enriched clocks. This unbiased approach without a strong focus on chronological age or any specific outcome, such as mortality, as well as the comprehensive evaluation of signatures across a variety of independent datasets, populations, and multiple species (human and mouse), are strengths of the study. Importantly, the modules described in the current study exhibit distinct behaviors over the course of cellular reprogramming, providing insights into the connection of age-related DNAme sites and reprogramming. We note that age acceleration and deceleration remain challenging to interpret. The purpose of the current study was not to develop optimal clocks. Instead, we anticipate that our benchmarking of functionally enriched clocks, including a comparison to commonly leveraged existing clocks, across several exposures and age-related pathologies can help to elucidate systemic changes occurring under these conditions. In the future, this may help to develop better clocks and/or understanding of epigenetic changes with aging and cancer.

Previous studies had identified CpG sites associated with senescence or proliferation using IlluminaMethylation27K or IlluminaMethylation450K beadchip arrays^6,11,53^, including some that did not provide detailed lists of senescence-associated sites. Here, we perform a comprehensive survey of sites associated with senescence and proliferation, combining several datasets, and validate their correlation with known genes associated with these two cellular processes (*CDKN2A* and *MKI67*, respectively). We provide a detailed list of these sites in our supplementary materials (Supplementary Data 5-7). Moreover, we further dissect whether these sites are associated with age, thus providing relevant additional information to existing literature.

The study’s limitations include small sample sizes for certain subsets. We note that the functionally enriched clocks constructed for this study are of exploratory nature. For instance, we only looked at CpGs overall associated with senescence and did not further refine by different senescence types or biomarkers. Moreover, while association of senescence- or proliferation-associated CpGs was validated by assessing the correlation with mRNA expression of *CDKN2A* (p16) and *MKI67* (Ki67) (Figs. S1, S2), two genes associated with senescence and proliferation, further validation of functional-associated sites could be beneficial. One particularly exciting aspect would be to study the functional consequences of altering age-related functional modules identified in this study via epigenetic editing, investigating alterations in gene expression, protein levels, and cellular functionality. Our finding should form the basis of further mechanistic studies to improve our understanding of biomarkers of aging, rather than be used as individual clocks themselves, although many of them offered predictive values for 10-year mortality in buccal and blood samples (e.g., PCGTpure, SENpure, AGE), prognostic value for neoadjuvant chemotherapy response (e.g., PCGTpure, SENpure), or early detection of cervical pre-cancer stages (e.g., AGEepi). Our study only investigated three key hallmarks of aging/cancer that were selected due to their strong association with the epigenome and carcinogenesis. While this study provides an initial exploration of functionally enriched epigenetic clocks associated with these hallmarks, future work may benefit from additional systematic exploration of the association of the epigenome with other hallmarks, in a similar way to Kabacik et al. exploration of the association of epigenetic age with molecular and cellular hallmarks of aging^54^. Another limitation is the identification of murine PCGT sites using human orthologues. However, previous research indicated a high degree of evolutionary conservation of polycomb group gene targets^55^, and genes we considered as PCGT genes also exhibited high propensity scores (see Methods section) in a previous study describing the prediction of murine PCGTs^35^.

We identify consistent patterns associated with aging that (i) are aggravated in cancer, (ii) can be modified by carcinogenic exposures, and (iii) are decreased by risk-reducing measures, providing exciting prospects for preventive efforts and epigenetic disease risk monitoring. Our study also identifies a timeline of the earliest epigenetic changes in the process to carcinogenesis due to the use of preneoplastic CIN samples derived from cervical cancer screening.

Importantly, our data indicate that within the same individual, cancer may elicit an acceleration of aging in the tissue at risk but deceleration of aging in other tissues; in the case of breast cancer, hormone-sensitive cervical epithelium exhibits reduced values in functionally enriched aging clocks compared to controls, a finding we observe both in human observational data and a mouse model of breast cancer, while buccal samples share directionalities with cancer tissue (Fig. 3l), in line with other recent work^51^. The mouse study also allowed us to study the impact of antiprogestin-based chemoprevention on epigenetic features in the tissue at risk (breast tissue) and surrogate tissues (cervix, blood). Our data showed that antiprogestins rescued the deceleration in cervical samples observed with cancer, and this rescue was associated with the effectiveness of the chemoprevention: the clock values of animals that were given antiprogestins and did not develop tumors were more similar to controls than those of animals that were given antiprogestins but did develop tumors (mPCGTgen, Fig. 4b). In line with data from humans, our data in the mouse model showed that blood tissue did not display significant acceleration, or deceleration, in individuals with tumors (Fig. 4b).

Our findings suggest that aging features in hormone-sensitive surrogate tissues could represent a suitable means to monitor the effectiveness of cancer-preventive measures, but also raise questions about the potential interpretation of a deceleration of epigenetic clocks in such surrogate tissues: a deceleration of epigenetic age is typically broadly interpreted as rejuvenation and an improvement of health, whereas our findings indicate a deceleration in certain age modules of cervical samples may be associated with an increased breast cancer risk. While acceleration of various clocks in cancer tissue has been extensively studied and reported (e.g., in breast^46,56^), and our findings largely agree with this, the relative reduction in epigenetic aging features in certain surrogate tissues also stands in contrast to previous studies that consistently reported an acceleration of epigenetic age in surrogate tissues of patients with current or future cancers compared to controls (i.e., the most commonly studied surrogate—blood)^57–60^. We note that our analysis is based on a population average, i.e., comparing predicted ages of controls to those of cases after accounting for chronological age differences within each tissue. Thus, it is possible that many tissues showed age accelerations, yet these were not solely driven by cancer but also by different predicted ages in tissue in general. It is noteworthy that different cancer types exhibit different Horvath/other epigenetic age patterns.

The relative reduction in epigenetic age of surrogate tissues from cancer patients compared to controls will need to be further considered when developing markers to track epigenetic aging in surrogate tissues, as it is currently not clear how a deceleration of aging in such surrogate tissues should accordingly be interpreted with respect to cancer risk. It is possible that quantifying aging biomarkers across multiple tissues from the same individual may harbor more information with respect to organismal health and cancer risk. It is currently unclear what causes the variable effect of cancer on aging in various tissues (i.e., acceleration in cancer tissue but deceleration in surrogate tissues) observed in Figs. 3 and 4. One hypothesis previously proposed is that the epigenome of relevant surrogate tissues may be able to capture lifetime risk for a cancer, but it is currently unclear how this would result in a deceleration of aging trajectory. It is also possible that epigenetic alterations in surrogate tissue are caused by presence of a cancer in another tissue. Future studies longitudinally tracking epigenetic aging across multiple tissue and sample types, including preceding cancer, are required to improve our understanding of tissue-specific versus systemic (epigenetic) aging and disease risk.

## Supplementary information

Supplementary Information

Description of Additional Supplementary Files

Supplementary Data 1

Supplementary Data 2

Supplementary Data 3

Supplementary Data 4

Supplementary Data 5

Supplementary Data 6

Supplementary Data 7

Supplementary Data 8

Supplementary Data 9

Supplementary Data 10

Supplementary Data 11

Supplementary Data 12

Supplementary Movie 1

Supplementary Movie 2

Reporting summary

## Data Availability

Data used in this study can be accessed from NCBI Gene Expression Omnibus (GSE54848, GSE211668, GSE197512, GSE184159, GSE131280, GSE112812, GSE112873, GSE91069, GSE116375, GSE82234, GSE116375, GSE197512, GSE236260, GSE236260), The Cancer Genome Atlas, or the European Genome-Phenome Archive (EGAS00001005070, EGAS00001005078, EGAS00001005055, EGAS00001005033, EGAS00001005045), with the exception of data from the NSHD MRC 1946 birth cohort due to data privacy. NSHD MRC1946 methylation and clinical data are available to bona fide researchers upon request to the Data Sharing Committee via a standard application procedure. Further details can be found at https://skylark.ucl.ac.uk/NSHD/doku.php?id=home. Data underlying figures and tables for this manuscript are found in the Supplementary Source Data: Source data for Fig. 2a–c are provided in Supplementary Data 8, 9, and 10, respectively. Source Data for Fig. 3 are provided in Supplementary Data 11. Source data for Fig. 4b are provided in Supplementary Data 12.

## Code availability

Code to fully reproduce the findings in this study is provided under github.com/chiaraherzog/functional_aging_sigs/^36^.
