# Supplementary material for: Functionally enriched epigenetic clocks reveal tissue-specific discordant aging patterns in individuals with cancer
Source: Commun Med (Lond). 2025 Apr 2;5:98. doi: 10.1038/s43856-025-00739-4 (PMC11965555; doi:10.1038/s43856-025-00739-4)
Supplement: Supplementary file 1 — Supplementary Information [file 43856_2025_739_MOESM1_ESM.pdf]

Functionally enriched epigenetic clocks reveal tissue-specific discordant aging patterns in individuals with cancer

Supplementary Information

Chiara Herzog, Elisa Redl, James Barrett, Sepideh Aminzadeh-Gohari, Daniela Weber, Julia Tevini, Roland Lang, Barbara Kofler, and Martin Widschwendter

Contents

1 Figure S1. Identification of senescence-associated CpGs. 2

2 Figure S2. Identification of proliferation-associated CpGs. 3

3 Figure S3. Representative examples of CpG sites passing cell type-specific criteria. 4

4 Figure S4. Overlaps of age-, proliferation-, senescence-, and PCGT-associate CpGs. 4

5 Figure S5. Association of new clocks with age. 6

6 Figure S6. Principal component analysis of mouse tissue DNAm data. 7

7 Figure S7. Representative AUCs for clock values higher or lower than in reference group in the TCGA-BRCA set. 8

8 Figure S8. Idenfication of PCGT CpG sites in mice and age-related CpGs in BALB/c mice. 9

9 Supplementary Data 1. Datasets and accession numbers used in this study. 11

10 Supplementary Data 2. CpG Overlap criteria. 11

11 Supplementary Data 3. Reference and comparison groups in Figure 3. 11

12 Supplementary Data 4. Overview of CpGs in age signatures defined in the current study. 11

13 Supplementary Data 5. CpGs associated with senescence in the current study. 11

14 Supplementary Data 6. CpGs associated with proliferation in the current study. 11

15 Supplementary Data 7. PCGT-associated CpGs in the current study. 11

16 Supplementary Data 8. Source data for Figure 2a. 11

17 Supplementary Data 9. Source data for Figure 2b. 11

18 Supplementary Data 10. Source data for Figure 2c. 11

19 Supplementary Data 11. Source data for Figure 3. 11

20 Supplementary Data 12. Source data for Figure 4b. **11**

21 Supplementary Movie 1. Animation to explain positive AUC based on thresholds. **11**

22 Supplementary Movie 2. Animation to explain a negative AUC based on thresholds. **11**

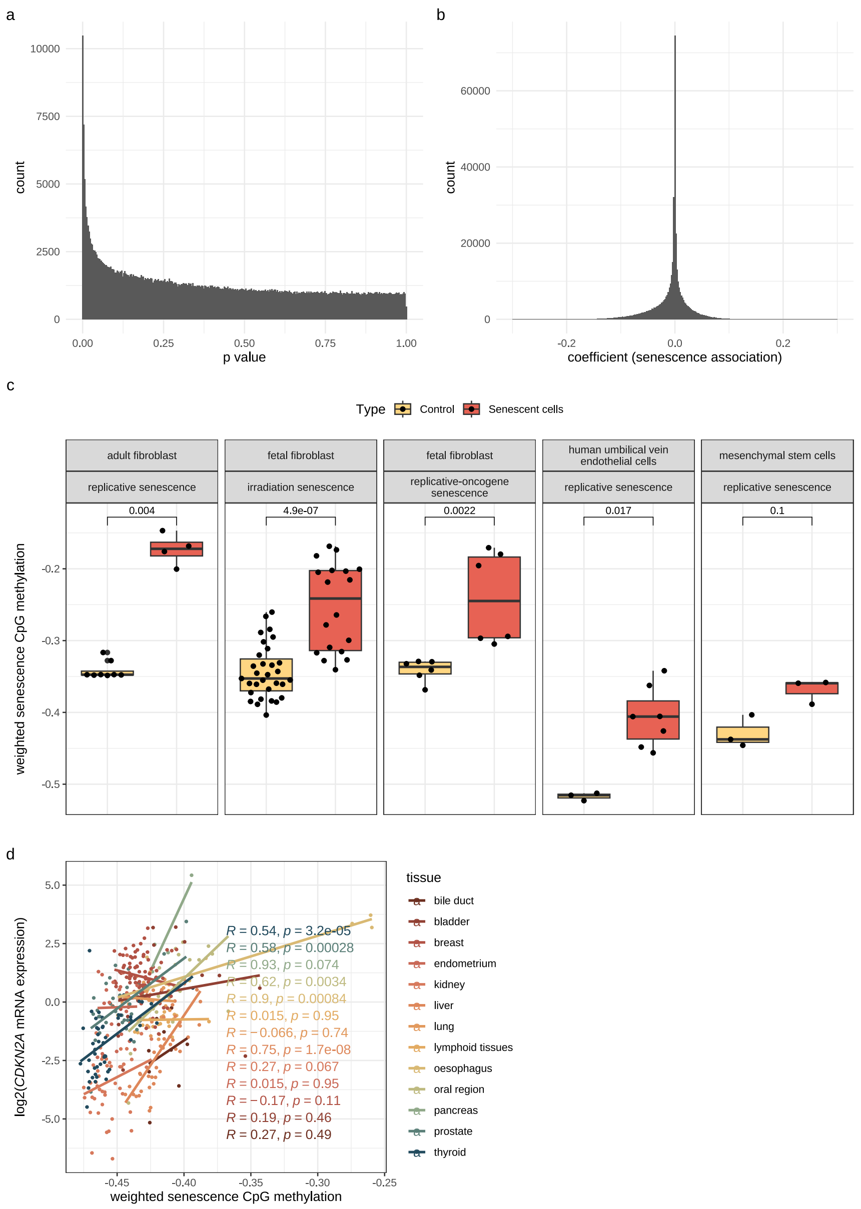

Figure S1. Identification of senescence-associated CpGs. **a**Histogram of p values for differential methylation analysis of senescence status, accounting for dataset and cell type. **b** Coefficient for association with methylation value by time reveals more loss than gain in methylation with senescence. **c** The weighted senescence methylation score is elevated in all senescence datasets used for development of the signature. Boxplot indicates median and interquartile range with overlaid individual data points. P values were computed comparing control and senescent cells using a two-sided Wilcoxon test. **d** The weighted senescence methylation score is positively correlated with mRNA expression of the key senescence-associated gene *CDKN2A* (p16) in 5/13 with $p < 0.05$ evaluated normal tissues with matched methylation and mRNA expression data from TCGA (individual data points are plotted). Correlation coefficient is Pearson's rho.

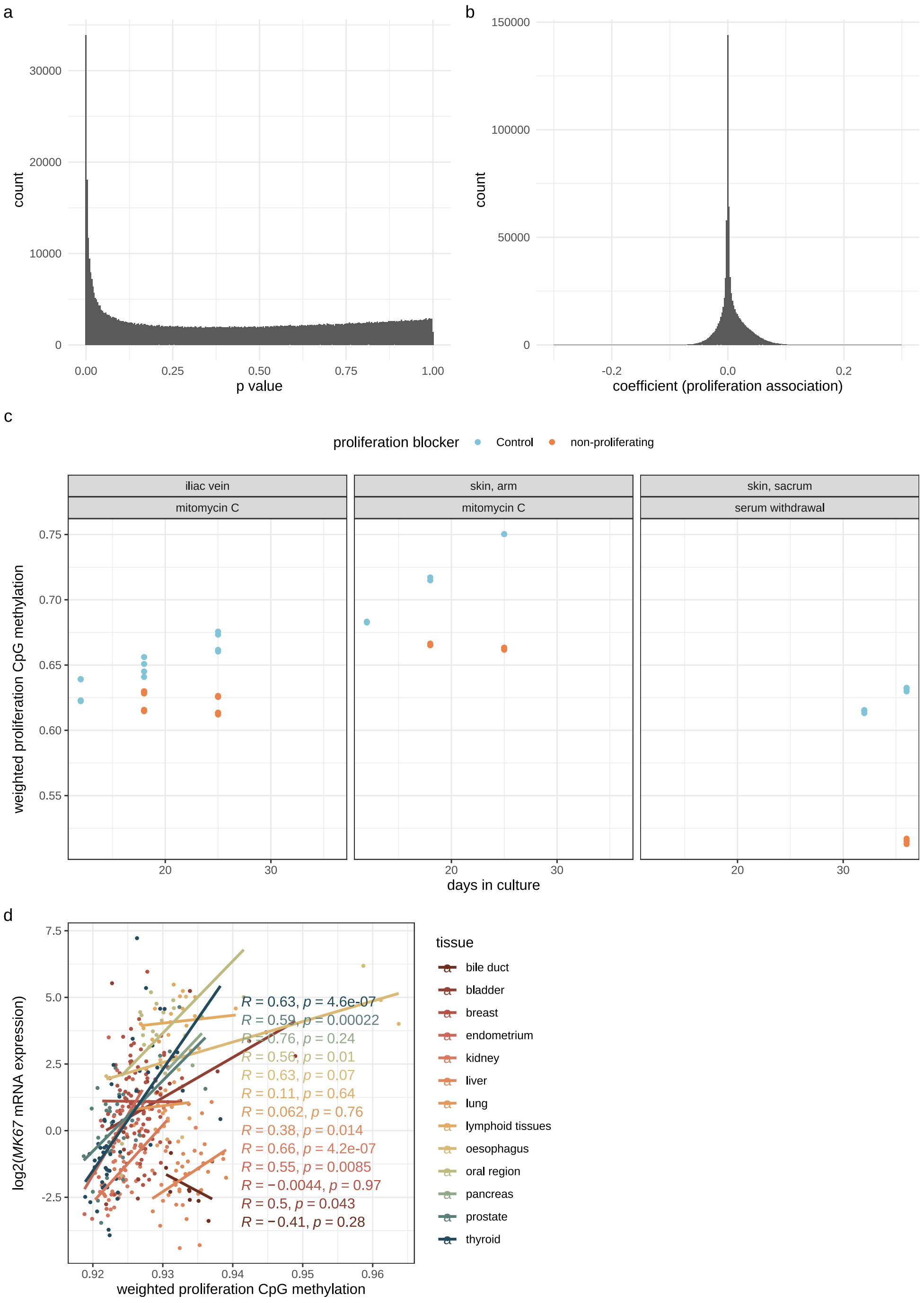

Figure S2. Identification of proliferation-associated CpGs. a Histogram of p values for differential methylation analysis of (reduced) proliferation status, accounting for tissue type and subexperiment (mitomycin C or serum withdrawal). **b** Coefficient for association with methylation value by time reveals more gain than loss of methylation with reduced proliferation **c** The weighted proliferation methylation score is consistently reduced with reduced proliferation across subexperiments in the dataset used to identify sites. **D** The weighted proliferation methylation score is positively correlated with mRNA expression of the key proliferation-associated gene *MKI67* (ki67) in 9/13 evaluated normal tissues with matched methylation and mRNA expression data from TCGA. Correlation coefficient is Pearson's rho.

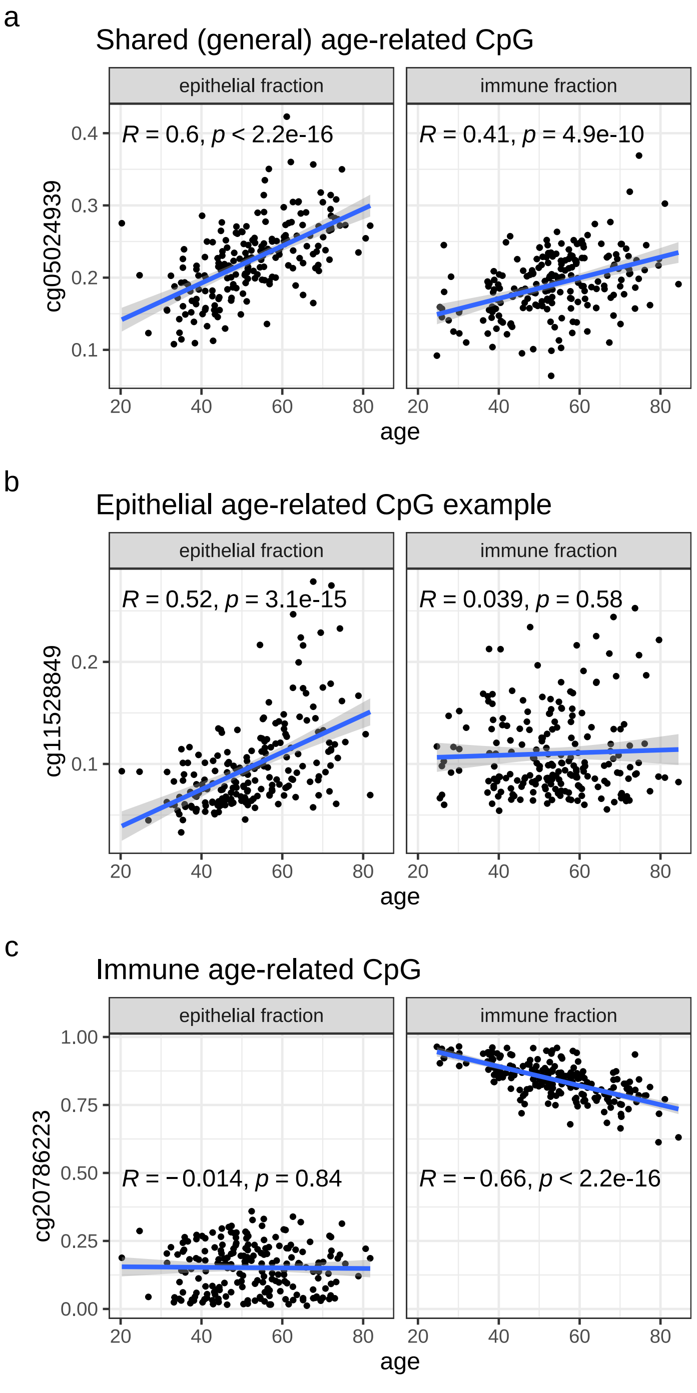

Figure S3. Representative examples of CpG sites passing cell type-specific criteria. a CpG correlated with chronological age in both epithelial (cervical and buccal samples with less than 20% inferred immune cell proportion) and immune cells (blood), **b** only epithelial cells, or **c** only immune cells. Correlation coefficient is Pearson's rho.

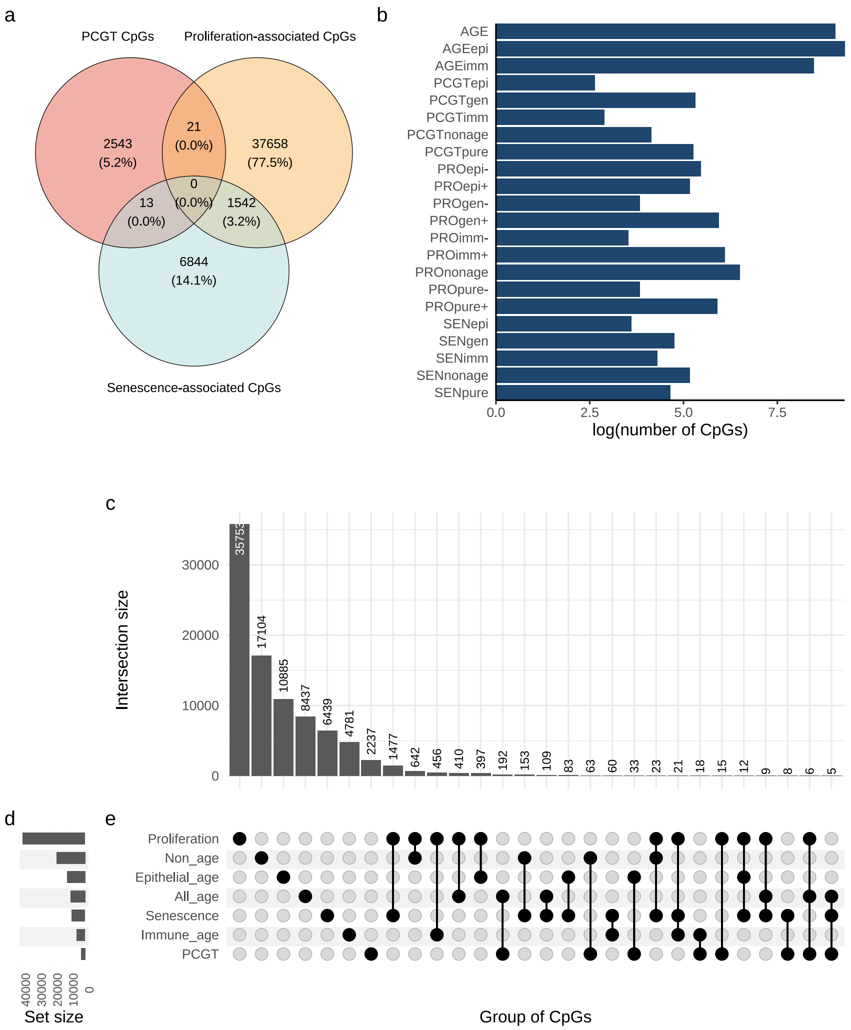

Figure S4. Overlaps of age-, proliferation-, senescence-, and PCGT-associate CpGs. a Venn diagram of CpGs associated with senescence, proliferation (both at FDR-adjusted p<0.05), or PCGTs. **b** Size of Clocks (number of CpGs). **c** UpSet plot of age-, proliferation-, senescence, and PCGT-associated CpGs.

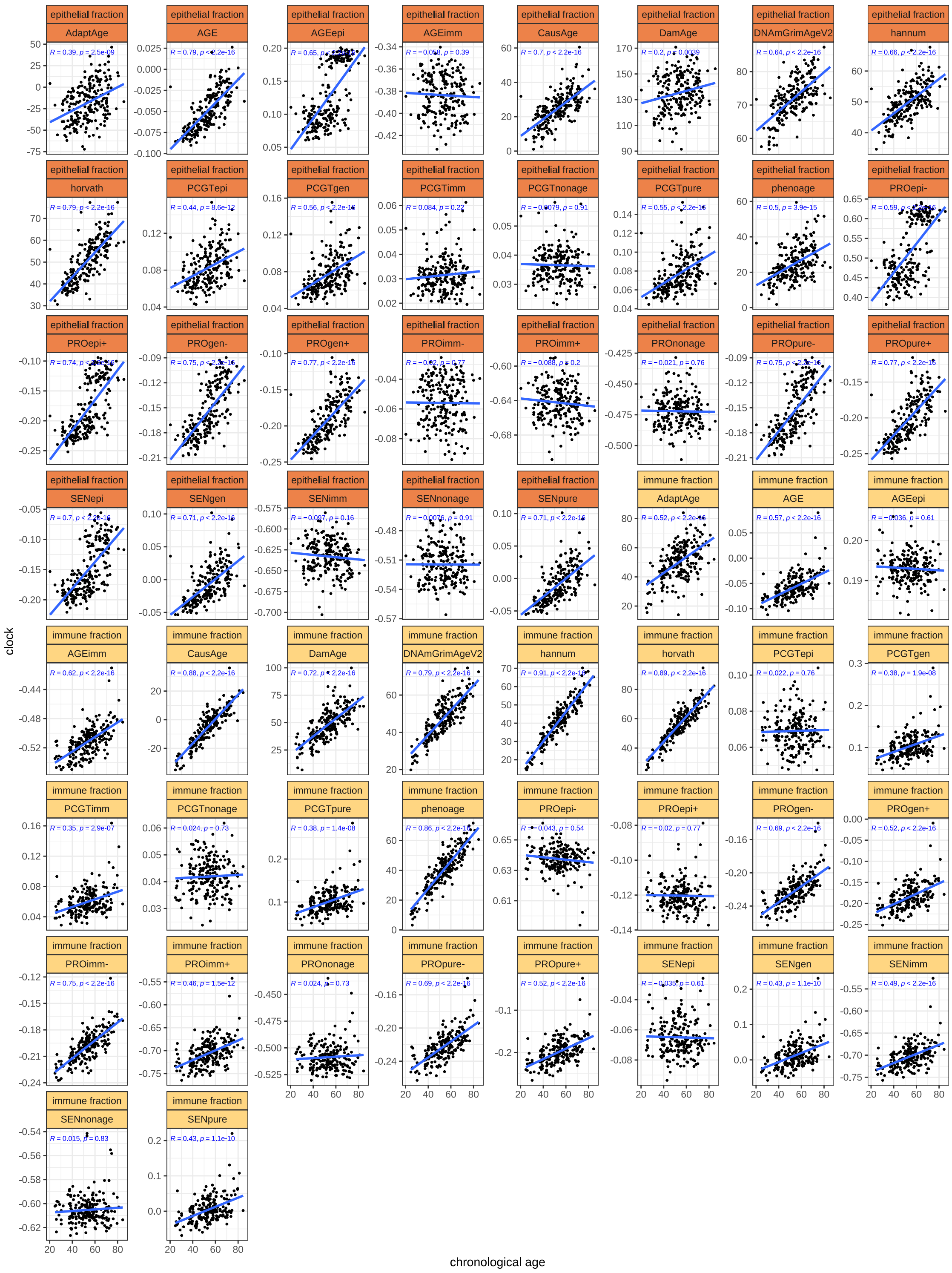

Figure S5. Correlation of clocks with age. Clocks were correlated with age in epithelial cells (cervical or buccal samples with an inferred immune cell proportion of less that 20%) or immune cells (blood samples) used to derive the signatures. Correlation coefficient is Pearson's rho.

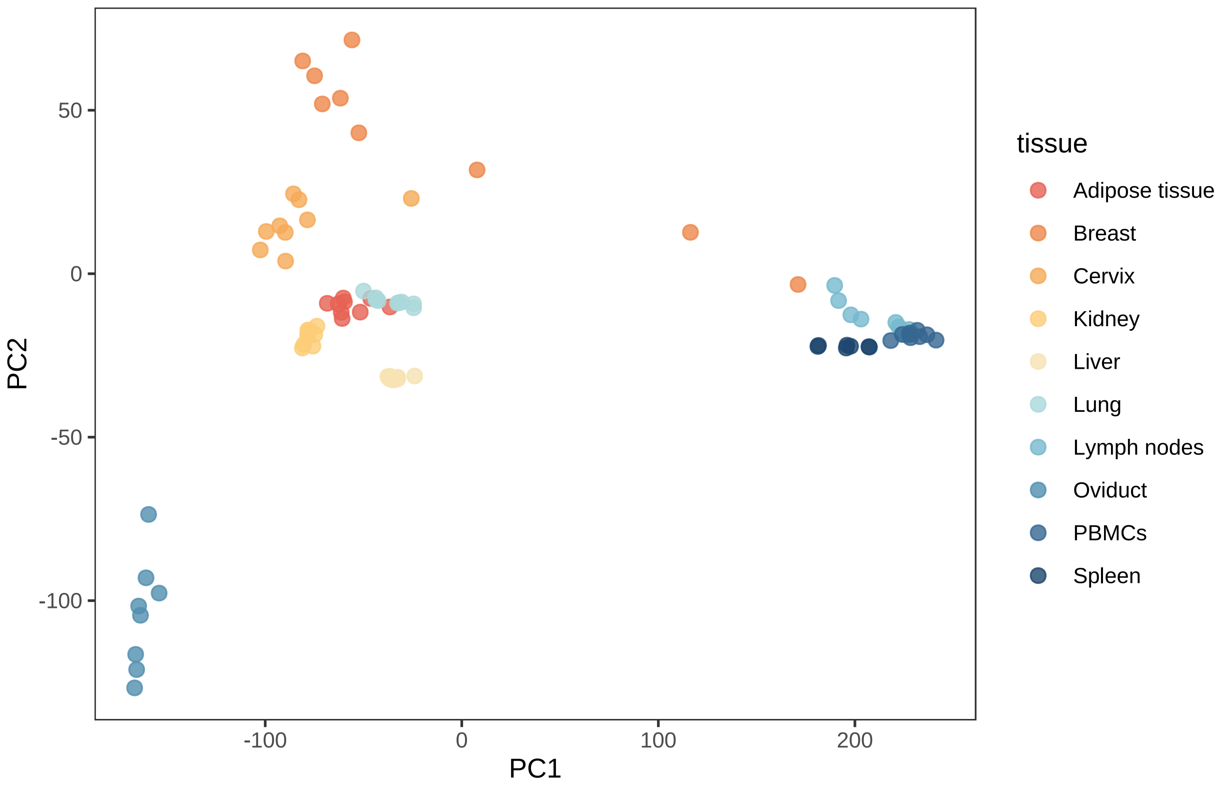

Figure S6. Principal component analysis of mouse tissue DNAm data. The first two principal components of the top 30,000 variable CpGs are shown for the age study.

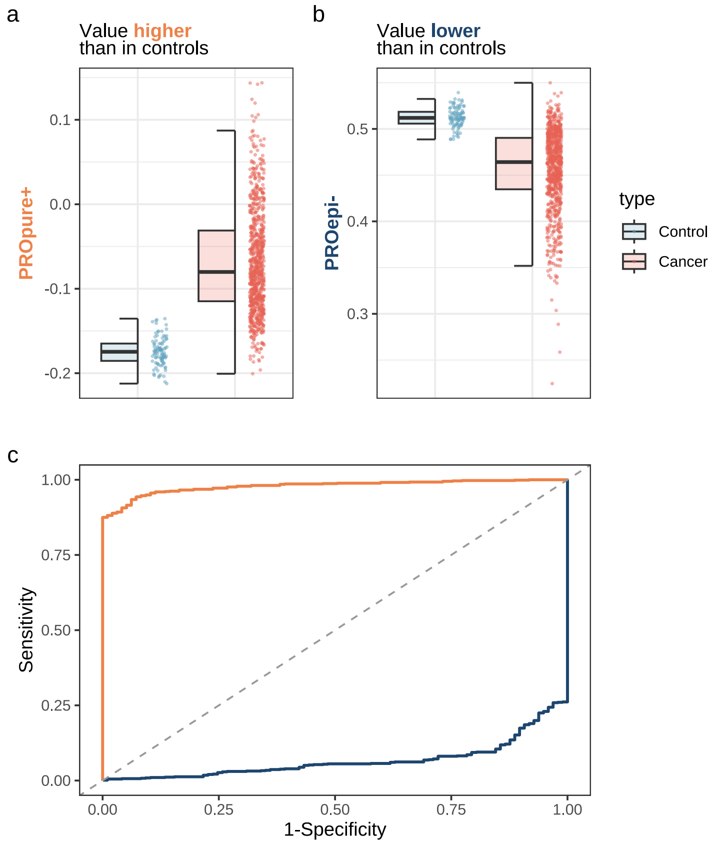

Figure S7. Representative AUCs for clock values higher or lower than in reference group in the TCGA-BRCA set. a PROpure- exhibits higher valus in the comparison group (cancer tissue) than in the reference group (control tissue), whereas **b** PROepi- exhibits lower values of the clock in the comparison group (cancer tissue) than in the reference group (control tissue). **c** AUC diagram illustrating high and low values. An AUC value of 0.5 would indicate no distinction.

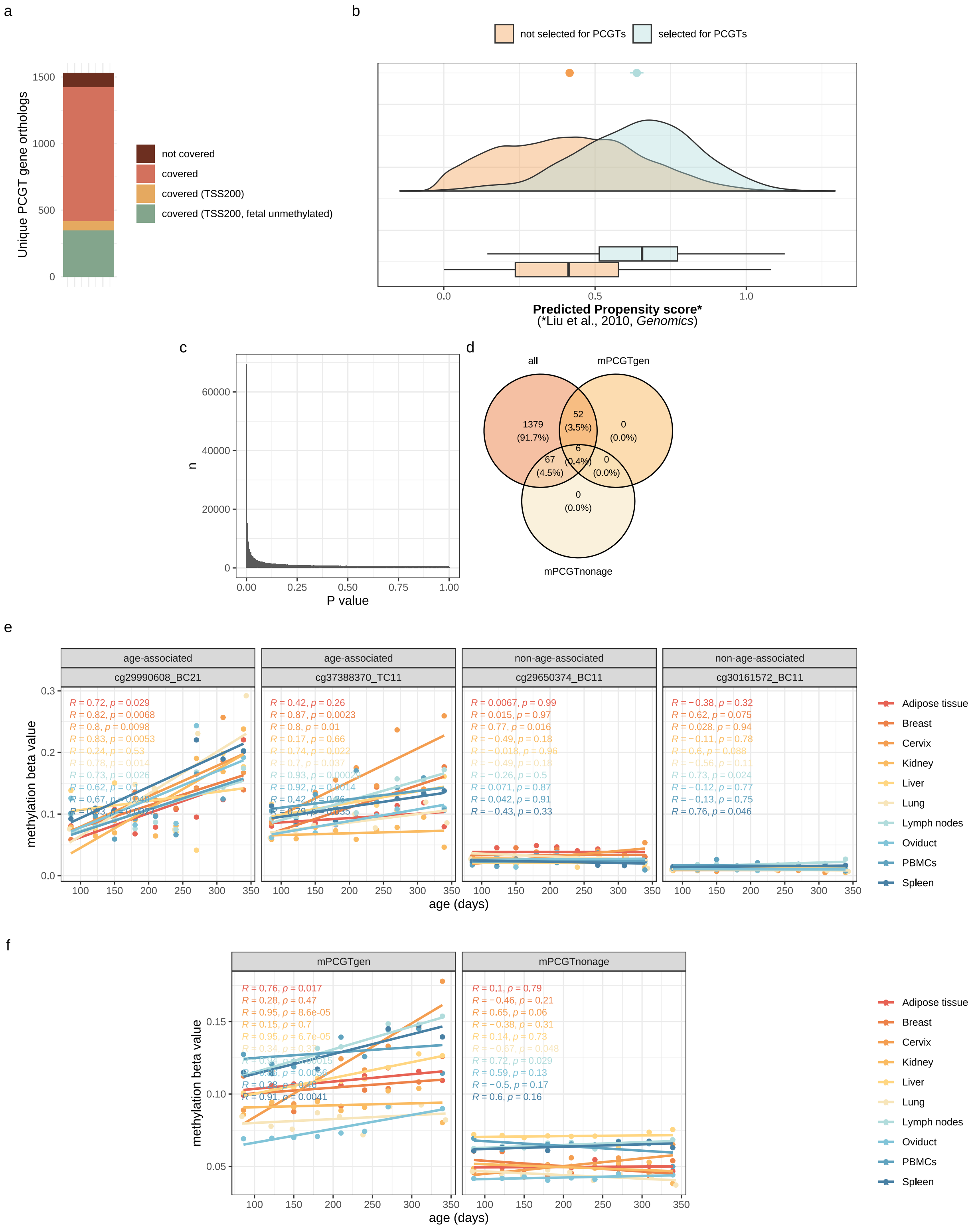

Figure S8. Idenfication of PCGT sites and age-related CpGs in BALB/c mice. a Unique murine PCGT orthologs and their coverage on the mouse methylation array. **b** Plot of the Liu *et al.* propensity score of genes that were selected as PCGT genes or not selected as PCGT genes in the current study. **c** P value histogram for association of age with beta methylation levels after correcting for tissue type. **d** Overlap of all

unique PCGT orthologs covered on the mouse methylation array with sites covered in age-related mouse PCGTgen (mPCGTgen) or non-age-related mPCGTnonage sites. **e** Methylation levels in various tissues in example sites for age-associated or non-age-associated CpGs covered in mPCGTgen and mPCGTnonage, respectively. **F** Mean methylation levels in various tissues for mPCGTgen and mPCGTnonage index.

Legends for supplementary data and movies

Supplementary Data 1. Datasets and accession numbers used in this study.

Supplementary Data 2. CpG Overlap criteria.

Supplementary Data 3. Reference and comparison groups in Figure 3.

Supplementary Data 4. Overview of CpGs in age signatures defined in the current study.

Supplementary Data 5. CpGs associated with senescence in the current study.

Supplementary Data 6. CpGs associated with proliferation in the current study.

Supplementary Data 7. PCGT-associated CpGs in the current study.

Supplementary Data 8. Source data for Figure 2a.

Supplementary Data 9. Source data for Figure 2b.

Supplementary Data 10. Source data for Figure 2c.

Supplementary Data 11. Source data for Figure 3.

Supplementary Data 12. Source data for Figure 4b.

Supplementary Movie 1. Animation to explain positive AUC based on thresholds. See additional .gif file.

Supplementary Movie 2. Animation to explain a negative AUC based on thresholds. See additional .gif file.
