# Supplementary material for: Functionally enriched epigenetic clocks reveal tissue-specific discordant aging patterns in individuals with cancer
Source: Commun Med (Lond). 2025 Apr 2;5:98. doi: 10.1038/s43856-025-00739-4 (PMC11965555; doi:10.1038/s43856-025-00739-4)
Supplement: Supplementary file 2 — Description of Additional Supplementary Files [file 43856_2025_739_MOESM2_ESM.pdf]

# Description of Additional Supplementary Files

**File name:** Supplementary Data 1.

**File description:** Datasets and accession numbers used in this study.

**File name:** Supplementary Data 2.

**File description:** CpG Overlap criteria.

**File name:** Supplementary Data 3.

**File description:** Reference and comparison groups in Figure 3.

**File name:** Supplementary Data 4.

**File description:** Overview of CpGs in age signatures defined in the current study.

**File name:** Supplementary Data 5.

**File description:** CpGs associated with senescence in the current study.

**File name:** Supplementary Data 6.

**File description:** CpGs associated with proliferation in the current study.

**File name:** Supplementary Data 7.

**File description:** PCGT-associated CpGs in the current study.

**File name:** Supplementary Data 8.

**File description:** Source data for Figure 2a.

**File name:** Supplementary Data 9.

**File description:** Source data for Figure 2b.

**File name:** Supplementary Data 10.

**File description:** Source data for Figure 2c.

**File name:** Supplementary Data 11.

**File description:** Source data for Figure 3.

**File name:** Supplementary Data 12.

**File description:** Source data for Figure 4b.

**File name:** Supplementary Movie 1.

**File description:** Animation to explain positive AUC based on thresholds. See additional .gif file.

**File name:** Supplementary Movie 2.

**File description:** Animation to explain a negative AUC based on thresholds. See additional .gif file.
