# Supplementary figures and images for: Functionally enriched epigenetic clocks reveal tissue-specific discordant aging patterns in individuals with cancer
Source: Commun Med (Lond). 2025 Apr 2;5:98. doi: 10.1038/s43856-025-00739-4 (PMC11965555; doi:10.1038/s43856-025-00739-4)

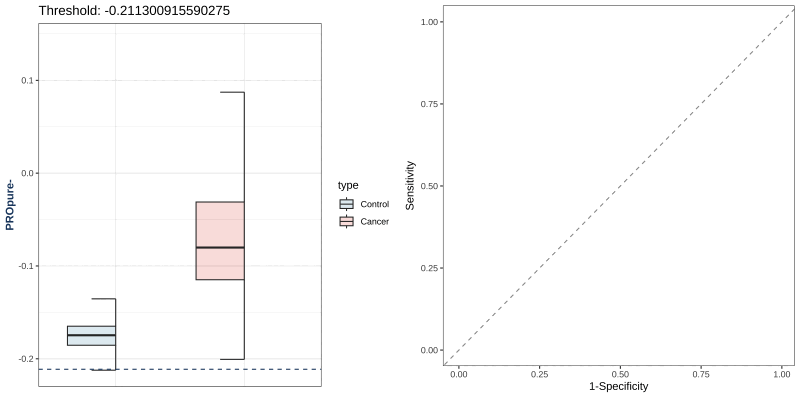

Supplement: Supplementary file 15 — Supplementary Movie 1 [file 43856_2025_739_MOESM15_ESM.gif]

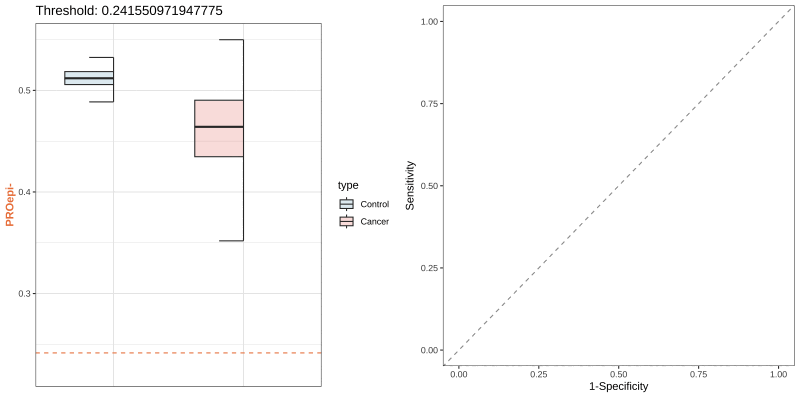

Supplement: Supplementary file 16 — Supplementary Movie 2 [file 43856_2025_739_MOESM16_ESM.gif]
