# Supplementary material for: Functionally enriched epigenetic clocks reveal tissue-specific discordant aging patterns in individuals with cancer
Source: Commun Med (Lond). 2025 Apr 2;5:98. doi: 10.1038/s43856-025-00739-4 (PMC11965555; doi:10.1038/s43856-025-00739-4)
Supplement: Supplementary file 17 — Reporting summary [file 43856_2025_739_MOESM17_ESM.pdf]

nature portfolio

Corresponding author(s): Martin Widschwendter

Last updated by author(s): Nov 9, 2024

Reporting Summary

Nature Portfolio wishes to improve the reproducibility of the work that we publish. This form provides structure for consistency and transparency in reporting. For further information on Nature Portfolio policies, see our Editorial Policies and the Editorial Policy Checklist.

Statistics

For all statistical analyses, confirm that the following items are present in the figure legend, table legend, main text, or Methods section.

n/a	Confirmed	
☐	☒	The exact sample size (*n*) for each experimental group/condition, given as a discrete number and unit of measurement
☐	☒	A statement on whether measurements were taken from distinct samples or whether the same sample was measured repeatedly
☐	☒	The statistical test(s) used AND whether they are one- or two-sided<br>*Only common tests should be described solely by name; describe more complex techniques in the Methods section.*
☐	☒	A description of all covariates tested
☐	☒	A description of any assumptions or corrections, such as tests of normality and adjustment for multiple comparisons
☐	☒	A full description of the statistical parameters including central tendency (e.g. means) or other basic estimates (e.g. regression coefficient) AND variation (e.g. standard deviation) or associated estimates of uncertainty (e.g. confidence intervals)
☐	☒	For null hypothesis testing, the test statistic (e.g. *F*, *t*, *r*) with confidence intervals, effect sizes, degrees of freedom and *P* value noted<br>*Give P values as exact values whenever suitable.*
☒	☐	For Bayesian analysis, information on the choice of priors and Markov chain Monte Carlo settings
☒	☐	For hierarchical and complex designs, identification of the appropriate level for tests and full reporting of outcomes
☐	☒	Estimates of effect sizes (e.g. Cohen's *d*, Pearson's *r*), indicating how they were calculated

Our web collection on statistics for biologists contains articles on many of the points above.

Software and code

Policy information about availability of computer code

Data collection	/
Data analysis	All analyses in this manuscript were conducted using the open source statistics software R. The full code for analyses is provided here: github.com/chiaraherzog/functional_aging_sigs/. A DOI is provided, too.

For manuscripts utilizing custom algorithms or software that are central to the research but not yet described in published literature, software must be made available to editors and reviewers. We strongly encourage code deposition in a community repository (e.g. GitHub). See the Nature Portfolio guidelines for submitting code & software for further information.

Data

Policy information about availability of data

All manuscripts must include a data availability statement. This statement should provide the following information, where applicable:

- Accession codes, unique identifiers, or web links for publicly available datasets
- A description of any restrictions on data availability
- For clinical datasets or third party data, please ensure that the statement adheres to our policy

Data used in this study can be accessed from NCBI Gene Expression Omnibus (GSE54848, GSE211668, GSE197512, GSE184159, GSE131280, GSE112812, GSE112873, GSE91069, GSE116375, GSE82234, GSE116375, GSE197512, GSE236260, GSE236260), The Cancer Genome Atlas, or the European Genome-Phenome Archive (EGAS00001005070, EGAS00001005078, EGAS00001005055, EGAS00001005033, EGAS00001005045), with the exception of data from the NSHD MRC 1946

birth cohort due to data privacy. NSHD MRC1946 methylation and clinical data are available to bona fide researchers upon request to the Data Sharing Committee via a standard application procedure. Further details can be found at https://skylark.ucl.ac.uk/NSHD/doku.php?id=home.
Data underlying figures and tables for this manuscript are found in the Source Data.

Human research participants

Policy information about studies involving human research participants and Sex and Gender in Research.

Reporting on sex and gender	Some sex-specific analyses were performed were relevant, e.g. for female-specific cancers and sample collection.
Population characteristics	This study leverages several previously described datasets and a new mouse methylation study. A full list of datasets, including accession numbers for GEO datasets, is provided in Supplementary Data 1.
Recruitment	N/a
Ethics oversight	N/a

Note that full information on the approval of the study protocol must also be provided in the manuscript.

Field-specific reporting

Please select the one below that is the best fit for your research. If you are not sure, read the appropriate sections before making your selection.

☒ Life sciences ☐ Behavioural & social sciences ☐ Ecological, evolutionary & environmental sciences

For a reference copy of the document with all sections, see nature.com/documents/nr-reporting-summary-flat.pdf

Life sciences study design

All studies must disclose on these points even when the disclosure is negative.

Sample size	We did not perform a formal sample size calculation for this study.
Data exclusions	No data were excluded.
Replication	This study performed an analysis of existing datasets and no technical replicates were included.
Randomization	N/a
Blinding	N/a

Reporting for specific materials, systems and methods

We require information from authors about some types of materials, experimental systems and methods used in many studies. Here, indicate whether each material, system or method listed is relevant to your study. If you are not sure if a list item applies to your research, read the appropriate section before selecting a response.

Materials & experimental systems

n/a	Involved in the study	
☒	☐	Antibodies
☒	☐	Eukaryotic cell lines
☒	☐	Palaeontology and archaeology
☐	☒	Animals and other organisms
☒	☐	Clinical data
☒	☐	Dual use research of concern

Methods

n/a	Involved in the study	
☒	☐	ChIP-seq
☒	☐	Flow cytometry
☒	☐	MRI-based neuroimaging

Animals and other research organisms

Policy information about studies involving animals; ARRIVE guidelines recommended for reporting animal research, and Sex and Gender in Research

Laboratory animals	The study used 7-week-old female BALB/c mice.
Wild animals	n/a
Reporting on sex	Female mice only for this model
Field-collected samples	n/a
Ethics oversight	Animal experiments were performed at the animal facility of the Paracelsus Medical University Salzburg in accordance with the Austrian federal ministry of education, science and research (BMBWF), study approval No. 2021-0.236.530.

Note that full information on the approval of the study protocol must also be provided in the manuscript.
